# Environmental Orderliness Affects Self-Control and Creative Thinking: The Moderating Effects of Trait Self-Control

**DOI:** 10.3389/fpsyg.2020.01515

**Published:** 2020-07-31

**Authors:** Zhengyan Li, Ning Liu, Shouxin Li

**Affiliations:** School of Psychology, Shandong Normal University, Jinan, China

**Keywords:** environmental orderliness, self-control behaviors, creative thinking, trait self-control, self-control resources, order, disorder, persistence

## Abstract

Environmental orderliness can affect both self-control behaviors and creative thinking; however, little research has focused on the moderators of this effect. In this study, we investigated the moderating effect of trait self-control on environmental orderliness, which influences both state self-control behaviors and creative thinking. In Experiment 1, we explored whether trait self-control could moderate the effect of environmental orderliness on state behavioral self-control. The participants have been exposed to an orderly or a disorderly room and asked to complete a breath-holding task to measure self-control. The results showed that low trait self-control participants were more self-controlled in the orderly environment, whereas the self-control of those with high trait self-control was not affected by environmental orderliness. In Experiment 2, the moderating effect of trait self-control on environmental orderliness affecting creative thinking was investigated with a picture priming orderliness and the Alternative Uses Test. As expected, the participants with high trait self-control in the disorderly environment had better creative thinking performance, although there was no difference in the performance of those with low trait self-control between the two environmental orderliness conditions. The results demonstrated that trait self-control could moderate the dual effect of environmental orderliness. The present study sheds light on the effect of environmental orderliness and contributes to the understanding of the common mechanism of the dual effect; also, it has practical implications for the shaping and cultivation of individuals' self-control behaviors and creative thinking.

## Introduction

Environmental orderliness refers to the degree of order or disorder of the physical environment. As a feature of the physical environment, environmental orderliness has many psychological and behavioral consequences, such as immoral and illegal behavior (Keizer et al., 2008), confirmatory information processing (Niedernhuber et al., 2014), construal level, and global-local perceptual processing (Li et al., 2019a,b). Environmental orderliness has also been shown to affect behavioral self-control and creative thinking. Compared to a disorderly environment, an orderly environment, in which the items are arranged in a structured and ordered manner, is associated with maintaining order, abiding by norms, and more benefits to individuals' self-control (Fan et al., 2012; Vohs et al., 2013). Unexpectedly, however, a disorderly environment, which has long been considered to be associated with negative consequences, has also been shown to promote creative thinking (Chen et al., 2013; Vohs et al., 2013). Self-control and creative thinking play important roles in promoting individuals' development and social progress. Good self-control behaviors can help individuals achieve their goals and shape positive and socially desirable behaviors (Moffitt et al., 2011; De Ridder et al., 2012; Ning et al., 2015; Cao et al., 2018), thus creating a safe and orderly social atmosphere. Creative thinking is a necessary ability for creative activities. Through it, individuals can solve problems flexibly and creatively, produce new and valuable creative products, and promote social progress. The study of the influence of environmental orderliness on self-control and creative thinking does not only enrich the literature in the related fields; it may have also practical implications for the shaping and cultivation of individuals' self-control and creative thinking. Despite this, to date there has been no research on whether there are individual differences in the dual effect of environmental orderliness (environmental orderliness affects both self-control and creative thinking). Moreover, there is also little research on why these two seemingly unrelated dependent variables are both affected by environmental orderliness. Therefore, we aimed at answering these questions by focusing on the moderating effects of trait self-control on the dual effect of environmental orderliness.

Self-control refers to one's capacity to alter one's responses, especially to bring them into line with standards, such as one's ideas, values, and social expectations. It can enable an individual to start and maintain or stop and inhibit activities, such as making and implementing plans, resisting temptation, restraining impulses, and persisting in difficult tasks (Kopp, 1982; Baumeister et al., 2007). According to a resource model of self-control, all forms of self-control behaviors use common self-control resources, and these resources are limited. When previous self-control behaviors deplete resources and individuals are in a state of “ego depletion,” the remaining available resources will be reduced, and individuals will fail in subsequent state self-control behaviors (Baumeister and Heatherton, 1996; Baumeister et al., 1998, 2000). State self-control behaviors can be measured by tasks such as the breath-holding task (Vohs and Schmeichel, 2003) and unsolvable puzzles (Baumeister et al., 1998; Chae and Zhu, 2014). The resource model characterizes a person's capacity for self-control as a fluctuating state. However, self-control can also be considered as a stable personality trait, which may reflect different quantities of self-control resources, and individuals high in trait self-control have more self-control resources than those low in trait self-control (Baumeister, 2016). Trait self-control can be measured by a self-report scale, such as the Self-Control Scale (SCS), and it can predict a wide range of positive outcomes, such as positive academic performance (Zettler, 2011; King and Gaerlan, 2014; Honken et al., 2016; Cao et al., 2019), better relationships and interpersonal skills (Tangney et al., 2004; Vohs et al., 2011), and so on. Moreover, researchers have also found that trait self-control could influence state self-control behaviors in the laboratory; for example, high trait self-control individuals could perform better in a task requiring persistence than those with low trait self-control (Schmeichel and Zell, 2007).

As one of the influencing factors of self-control, an orderly environment has been shown to be more beneficial to individuals' state self-control behaviors than a disorderly environment. For example, some studies have shown that, compared to a disorderly environment, in an orderly one, individuals are less attracted by delicious but unhealthy food (Fan et al., 2012; Vohs et al., 2013), donate more money (Vohs et al., 2013), and prefer a larger reward available later (Fan et al., 2012). Chae and Zhu (2014) also showed that individuals in an orderly environment are less likely to buy on impulse and persist in an unsolvable task longer. Environmental orderliness seems to affect the individual's sense of personal control, further affecting the available self-control resources, and consequently influencing state self-control behaviors. Therefore, based on previous research findings, we expected that state self-control would improve in the order condition compared to the disordered one.

Creative thinking is a way of thinking that enables individuals to produce new and unique ideas or products (Sternberg and Lubart, 1996). Guilford (1967) indicated that creative thinking includes divergent and convergent thinking. Divergent thinking, as the core of creative thinking, refers to thinking in multiple directions and can reorganize current information and existing knowledge and experience to generate more unique and novel ideas (Guilford, 1956). The Alternative Uses Test (AUT) has widely been used as the main task to measure divergent thinking in previous studies (e.g., Friedman and Förster, 2001; Vohs et al., 2013; Wang et al., 2018).

The study of the relationship between environmental orderliness and creative thinking showed that disorder is more beneficial to creative thinking. Specifically, Vohs et al. (2013) demonstrated that individuals in a disorderly environment scored higher in overall creativity and average creativity and generated more creative ideas than those in an orderly one. Chen et al. (2013) showed that, in a disorderly environment, individuals not only performed better in fluency, flexibility, and originality, but also scored higher in the Remote Association Test and were more inclined to buy innovative products. In addition, other researchers found that individuals in a disorderly environment showed a higher prototype activation rate and correct rate of problem-solving, confirming once again the facilitating effect of disorder on creative thinking (Zheng et al., 2016). Moreover, researchers have preliminarily investigated how environmental orderliness influences creative thinking, a disorderly environment promotes creative thinking by improving cognitive flexibility and individuals become more inclined to think in a heuristic way (Chen et al., 2013). Therefore, based on previous research, we expected that creative thinking would improve in the disordered condition compared to the ordered one.

In sum, existing research has consistently shown that environmental order has a positive influence on state self-control behaviors, whereas environmental disorder is more beneficial to creative thinking. However, to our knowledge, there are still some questions to be answered, that is, are there individual differences in the dual effect of environmental orderliness? In addition, is there a common mechanism behind this dual effect? Studies of the respective psychological mechanisms of the dual effect showed that the perception of a threat to personal control mediates the relationship between environmental orderliness and state self-control behaviors (Chae and Zhu, 2014); cognitive flexibility and heuristic processing are the mechanisms by which environmental orderliness influences creative thinking (Chen et al., 2013). However, there seemed to be no connection between these two mechanisms. Other researchers tried to explain this dual effect from the perspective of a common mechanism. For example, Vohs et al. (2013), who first discussed the influence of environmental orderliness on both self-control and creative thinking, argued that environmental orderliness could change an individual's mind-set; an orderly environment is associated with valuing convention and tradition, thus it is more beneficial to self-control, whereas disorder, inspiring a break with convention, makes the individual produce more creative ideas. The explanation of the “world is random” model (WIR) for the dual effect was that through priming and increasing the judgment weight of randomness-related concepts, a disorderly environment decreases the sense of personal control and then leads to a subsequent failure of self-control. These changes may also reduce the motivation to exert executive control, facilitating the advancement into a flow state of unshackled creativity (Kotabe, 2014). However, the above researchers' speculation has not been confirmed by empirical data. To sum up, there has not been enough empirical research on whether there is a common mechanism of the dual effect of environmental orderliness and what the mechanism is.

We believe that available self-control resources are the key to this dual effect. Environmental orderliness influences the amount of available resources, which then affects state self-control behaviors and creative thinking. This was mainly supported by the following aspects. First, we found that the mechanisms previously mentioned, such as the sense of personal control, executive control, and heuristic processing, are closely related to self-control resources. Research has shown that the perception of a threat to personal control is related to ego depletion (Fischer et al., 2007). Self-control resources can affect the operation of executive control in the creative thinking process (Li, 2013). Moreover, individuals with self-control resource depletion tend to adopt a heuristic processing strategy rather than a systematic analytical processing strategy (Baumeister et al., 2008; Masicampo and Baumeister, 2008). Therefore, we argue that the available self-control resources are a more general mechanism behind the dual effect than these mechanisms. Second, it was found that environmental disorder depletes self-control resources (Chae and Zhu, 2014), and ego depletion affects both state self-control behaviors (Baumeister et al., 1998; Muraven et al., 1998; Vohs and Heatherton, 2000) and creative thinking (Chiu, 2014), which also provided support for our reasoning. Based on this, we believe that trait self-control, the variable that directly reflects the reserves of self-control resources, could affect the available resources, and then moderate the influence of environmental orderliness on self-control and creative thinking. Furthermore, if the moderating hypothesis was supported, it would demonstrate that the dual effect is based on the common mechanism of available self-control resources, which is reflected in the variable of trait self-control.

We assumed that trait self-control could moderate the influence of environmental orderliness on self-control. As mentioned before, trait self-control reflects one's reserves of resources, and previous studies have shown that, relative to low trait self-control individuals, high trait self-control people perform better in state self-control tasks that require more self-control resources (Schmeichel and Zell, 2007). In addition, a disorderly environment leads to a failure of state self-control via resource depletion (Chae and Zhu, 2014). Moreover, it has been shown that trait self-control moderates the impact of ego-depletion on state self-control behaviors, and those with more resources are less likely to be affected by resource depletion (Muraven et al., 2005; Dewall et al., 2007; Dvorak and Simons, 2009). Accordingly, we assumed that trait self-control moderated the effect of environmental orderliness on state self-control behaviors; specifically, an orderly environment would make low trait self-control individuals more self-controlled relative to a disorderly environment, whereas orderly or disorderly environments would lead to no difference in self-control in individuals with high trait self-control.

We also assumed that the influence of environmental orderliness on creative thinking might be moderated by trait self-control. Particularly, we were wondering if there was a compensation matching effect, that is, individuals with high and low trait self-control need a matching level of environmental orderliness to achieve the best overall effect of orderliness on self-control behaviors and creative thinking (low trait self-control and order; high trait self-control and disorder). Specifically, environmental order could be beneficial to state self-control only for the low trait self-control individuals, and environmental disorder could be beneficial to creative thinking only for the high trait self-control individuals. As mentioned earlier, the compensation matching between low trait self-control and environmental order was supported by previous literature; however, the literature evidence for the matching between high trait self-control and environmental disorder was more complex.

First, although there is no study that directly shows the influence of trait self-control on creative thinking, previous studies have shown that ego depletion could positively affect creative thinking (Chiu, 2014). In addition, we can also infer the influence of ego depletion on creative thinking from the perspective of the construal level. Construal level refers to the degree of abstraction that individuals use to represent information. High-level construal occurs when an individual represents information more abstractly, whereas low-level construal is beneficial for more concrete representations (Trope and Liberman, 2010). It has been shown that ego depletion leads individuals to be more inclined to use a low construal level (Bruyneel and Dewitte, 2012), and a low construal level is more unfavorable to creative thinking (Förster et al., 2004; Jia et al., 2009; Polman and Emich, 2011). Although the specific direction of the influence of ego depletion on creative thinking remains unclear, existing research has shown that resource depletion could affect creative thinking. Second, Chae and Zhu (2014) have shown that environmental disorder could deplete self-control resources. Hence, we assumed that trait self-control, reflecting the reserves of one's resources, could also moderate the impact of environmental orderliness on creative thinking.

In summary, to explore whether there were individual differences in the dual effect of environmental orderliness and identify the common mechanism of the dual effect, the present study tested the moderating role of trait self-control in the influence of environmental orderliness on both self-control and creative thinking. Specifically, our assumption was that an orderly environment would only increase state self-control behaviors in individuals with low trait self-control. Additionally, the influence of environmental orderliness on creative thinking could also be moderated by trait self-control, we expected that, if there was the compensation matching effect, environmental disorder could be beneficial to creative thinking only for the high trait self-control individuals, but not for the low trait self-control individuals.

**Figure 1:**
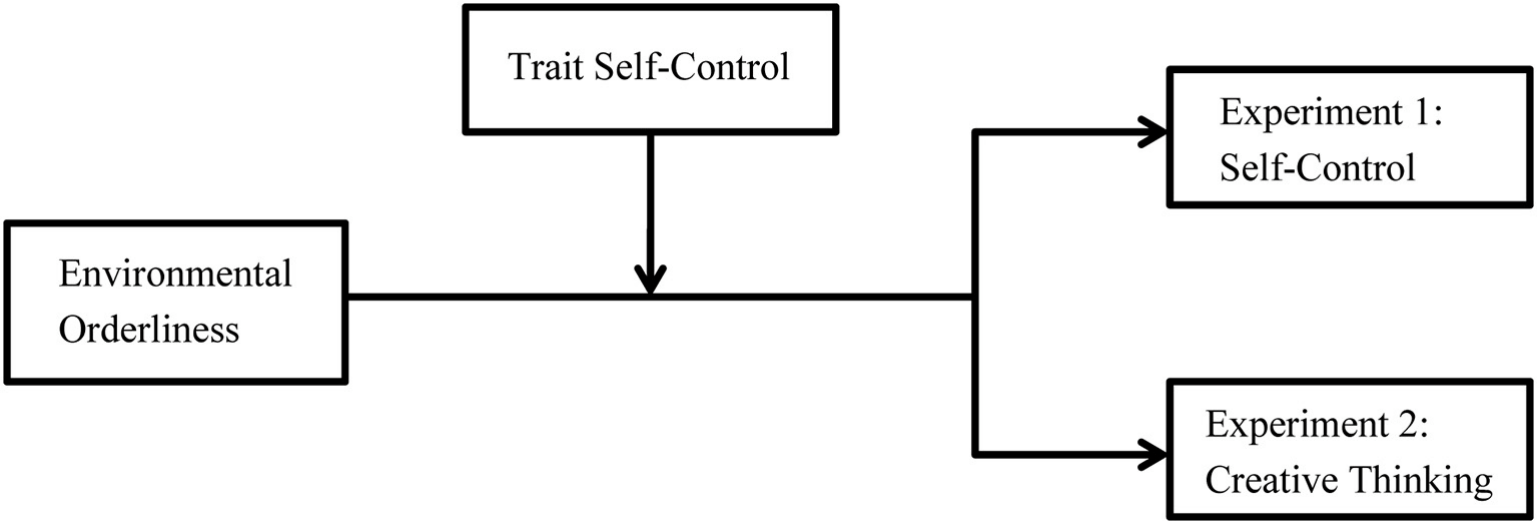
The moderating effects model of trait self-control on the dual effect of environmental orderliness.

These hypotheses were tested in two experiments. More specifically, in Experiment 1, we focused on the moderating effect of trait self-control on environmental orderliness affecting self-control by exposing participants to a real environment. In Experiment 2, its moderating effect on the influence of environmental orderliness on creative thinking was tested via a conceptual priming method. The moderating model tested in this study was shown in Figure 1. The present study provides a new research perspective for the environmental orderliness literature, contributes to the understanding of the common mechanism of the dual effect, and has practical implications for the shaping and cultivation of individuals' self-control behaviors and creative thinking.

## Experiment 1

Experiment 1 aimed to examine whether trait self-control would moderate the effect of environmental orderliness on state self-control behaviors. Self-control includes persistence and maintenance of responses (Vohs and Schmeichel, 2003). Many forms of self-control behaviors in daily life require individuals to overcome physical discomfort and test their physical and psychological endurance. For example, when holding their breath, individuals must try to restrain the body's demand for air. Hence, holding one's breath indicates perseverance of behavior. Therefore, in this experiment, a breath-holding task was chosen to measure self-control (Vohs and Schmeichel, 2003).

### Methods

#### Design and Participants

The experiment had a 2 (environmental order: order vs. disorder) ×2 (trait self-control: high self-control vs. low self-control) between-subjects design. The experiment was approved by the Institutional Review Board of the School of Psychology at the Shandong Normal University, and each participant signed the informed written consent form. A total of 171 college students filled out the Chinese version of the Brief SCS. A median split was used to sort participants into high vs. low self-control groups. One hundred and sixty-three participants (127 females; *M*_age_ = 19.80 years, *SD* = 1.07) volunteered to participate in the subsequent experiment, including 80 high trait self-control participants and 83 low trait self-control participants. The Brief SCS score of the high trait self-control group (*M*_high_ = 3.36, *SD* = 0.32) was higher than that of the low trait self-control group (*M*_low_ = 2.46, *SD* = 0.29), *t*_(161)_ = −19.04, *p* < 0.001, and Cohen's *d* = 2.98.

#### Procedure and Materials

Participants firstly completed the Chinese version of the Brief SCS to measure trait self-control (Tangney et al., 2004; Wang et al., 2015; Unger et al., 2016). They responded on a scale from 1 (not at all) to 5 (very much). Nine items were reverse coded, and the responses to the 13 items were then averaged to create a trait self-control score index (Cronbach's α = 0.83). The median split was used to sort participants into high vs. low self-control groups. The participants volunteered to take part in the follow-up experiment.

Upon arrival, participants were told that the experimenter was gathering baseline data on the average breath-holding capacity of college students in an attempt to find physiological markers for psychological states, and multiple measurements were needed to ensure the most reliable results. They were asked to hold their breath “for as long as you are able or until you have to give up,” and the experimenter timed the duration with a stopwatch (out of participants' views). They were measured twice before the manipulation of environmental orderliness as a baseline measurement of their performance. The mean time of these two measurements was designated Time 1 (Cronbach's α = 0.90).

**Figure 2:**
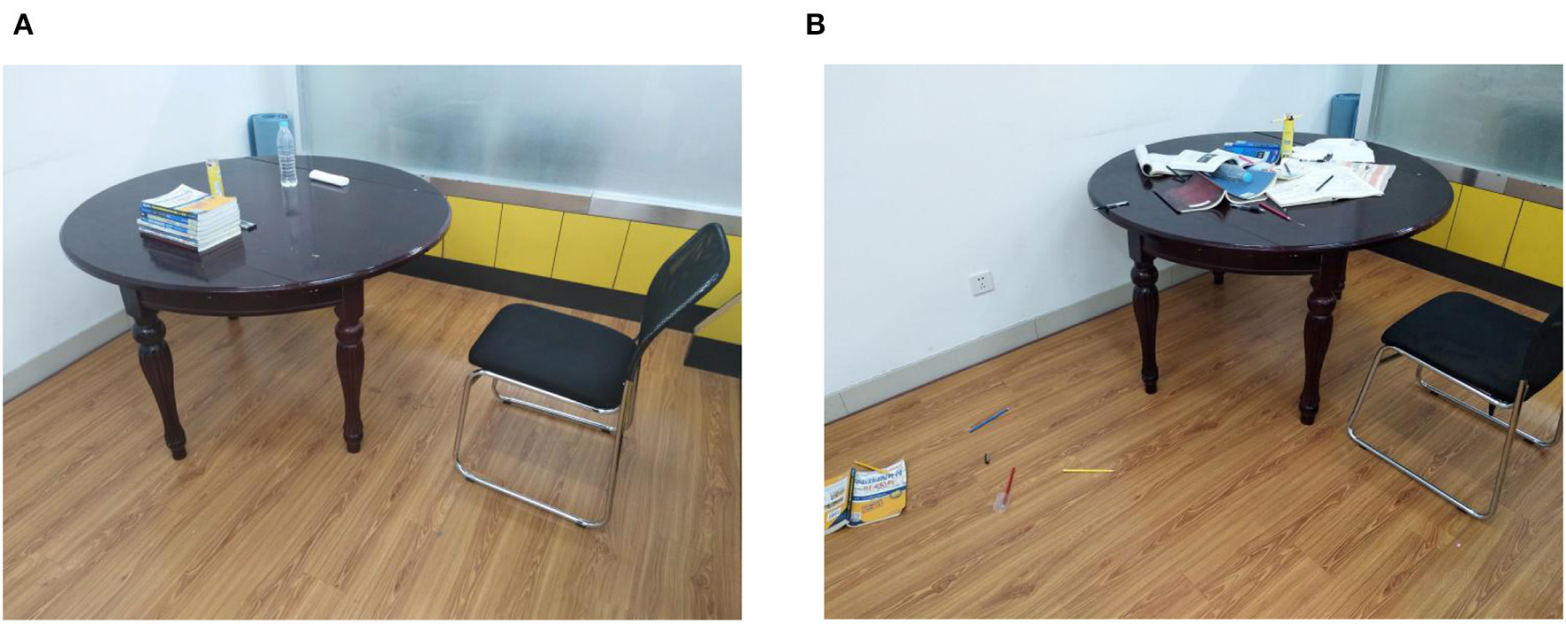
Illustration of the stimuli of Experiment 1: environmental order **(A)** and disorder **(B)** rooms.

Then, they were randomly assigned to one of the two environmental orderliness conditions. In the orderly room, books, pens, and other items were arranged in a structured and ordered manner on a table. In contrast, in the disorderly room, the same quantity of items was scattered on the table and on the floor (Figure 2). To fully expose the participants to the environment, they were asked to wait while the experimenter got the materials ready. The experimenter returned exactly 1 min later (Chae and Zhu, 2014), and then asked the participants to complete the 20-item Positive and Negative Affect Schedule (PANAS, Wang et al., 2015), which measured their current mood states as control variables (Cronbach's α was 0.84 for positive affect and 0.91 for negative affect), as the previous study showed that state self-control behavior was affected by emotions (Tice et al., 2007). Subsequently, participants were asked to perform the last two measurements as post-measurement after the manipulation. The mean time of these two measurements was designated Time 2 (Cronbach's α = 0.95).

Afterward, to assess whether our manipulation of environmental orderliness was successful, we asked two questions (i.e., “To what extent do you think this table is orderly? 1 = not at all, 7 = very orderly” and “How messy do you think this room is? 1 = not at all, 7 = very messy”). The second item was reverse coded, and the two items' scores were then averaged to create an environmental orderliness index (Cronbach's α = 0.91). We also measured the participants' daily living environmental orderliness as control variables with two questions “I usually keep the clothes orderly in my wardrobe” and “I am usually a person who pays great attention to environmental orderliness” on a 7-point scale (not at all to very much; Cronbach's α = 0.83) (Fan et al., 2012). The mean was used as the indicator. Finally, the participants were asked to guess the purpose of the experiment, fill in the demographic variables, and were debriefed and thanked.

### Results

SPSS 21.0 was used for data analysis. The data of 10 participants with mean differences in time exceeding ±3 standard deviations and one participant with an unfinished questionnaire were excluded from the final analysis. The final valid number was 152, including 36 high trait self-control participants and 40 low trait self-control participants in the orderly room, and 38 high trait self-control participants and 38 low trait self-control participants in the disorderly room. The mean difference in time under different conditions was analyzed.

#### Manipulation Check

An independent samples *t*-test performed on the perception of environmental orderliness showed that environmental orderliness was successfully manipulated, *t*_(150)_ = 25.33, *p* < 0.001, Cohen's *d* = 4.12. The participants in the orderly room perceived their workspace as more orderly (*M*_order_ = 6.03, *SD* = 0.84) than those in the disorderly one (*M*_disorder_ = 2.25, *SD* = 0.99).

#### The Breath-Holding Task

To eliminate interference of the baseline difference on the results, we first calculated difference scores by subtracting the baseline measurement (Time 1) from the post-measurement (Time 2). This difference score in breath-holding time from Time 1 to Time 2 measured the change in physical persistence and endurance as a function of the independent variables. Positive scores indicated an improvement in performance from Time 1 to Time 2, and the larger this positive score, the greater the improvement in participants' persistence from pre-measurement to post-measurement tasks; negative scores indicated a decline in performance (Muraven et al., 1998; Vohs and Schmeichel, 2003).

**Figure 3:**
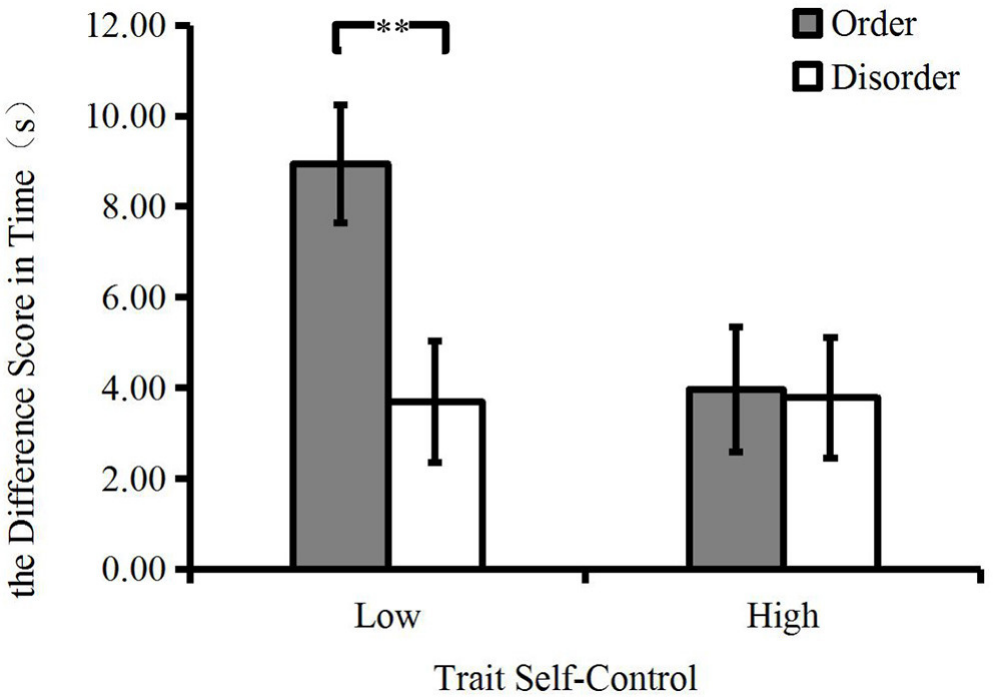
Experiment 1: participants' difference scores in time (error bars represent standard errors; ^**^p < 0.01).

We hypothesized that participants with low trait self-control in the orderly room would be more self-controlled than those in the disorderly room, whereas there would be no difference in the state self-control of participants with high trait self-control between the orderly and disorderly rooms. A two-way ANOVA was carried out on the difference scores in breath-holding time from Time 1 to Time 2. Our moderating prediction was supported by the marginally significant interaction between environmental orderliness and trait self-control, *F*_(1148)_ = 3.58, *p* = 0.06, $\eta_{p}^{2}$ = 0.02. As expected, for low trait self-control participants, the difference scores in time of those in the orderly room (*M*_order_ = 8.94, *SD* = 9.73) was larger than that of those in the disorderly room (*M*_disorder_ = 3.69, *SD* = 6.80), *F*_(1, 148)_ = 7.88, *p* = 0.006, $\eta_{p}^{2}$ = 0.05. However, for the participants with high trait self-control, there was no significant difference in the difference scores between the two environmental orderliness conditions (*M*_order_ = 3.96, *SD* = 4.72, *M*_disorder_ = 3.78, *SD* = 10.27), *F*_(1, 148)_ <1, *p* > 0.05 (see Figure 3). The results also showed that the difference scores in time of those in the orderly room (*M*_order_ = 6.58, *SD* = 8.12) was larger than that of those in the disorderly room (*M*_disorder_ = 3.74, *SD* = 8.65), *F*_(1, 148)_ = 4.10, *p* = 0.045, $\eta_{p}^{2}$ = 0.03. It was consistent with the main effect of environmental orderliness hypothesis. In addition, compared to the participants with high trait self-control (*M*_high_ = 3.87, *SD* = 8.01), those with low trait self-control (*M*_low_ = 6.39, *SD* = 8.78) had larger difference scores in breath-holding time, *F*_(1, 148)_ = 3.33, *p* = 0.07, $\eta_{p}^{2}$ = 0.02, which showed that low trait self-control participants' self-control performances improved more than high trait self-control participants from pre-measurement to post-measurement tasks.

#### Mood State and Daily Living Environmental Orderliness

A two-way ANOVA was carried out on the score of positive and negative affect and daily living environmental orderliness, respectively. The results showed that there was no significant main effects and interaction between environmental order and trait self-control on positive affect (*p*s > 0.05) or negative affect (*p*s > 0.05). Also, there was no significant main effect and interaction on daily living environmental orderliness (*p*s > 0.05). These results excluded the potential influence of mood state and daily living environmental orderliness on the dependent variables.

### Discussion

The findings from Experiment 1 showed that an orderly environment was more beneficial in improving participants' persistence in the breath-holding task, and more importantly, it revealed that trait self-control could moderate the influence of environmental orderliness on self-control. Specifically, for the participants with low trait self-control, the state self-control performances in the orderly environment were better than the disorderly environment. In contrast, for the participants with high trait self-control, there was no difference between the orderly and disorderly conditions, and consequently, the moderating model was supported (Figure 1). The results were consistent with previous findings showing that an orderly environment was more beneficial to state self-control behaviors than a disorderly one (Fan et al., 2012; Vohs et al., 2013; Chae and Zhu, 2014).

An interesting finding was that, compared with the disorderly environment, the orderly environment significantly improved the persistence of the participants with low trait self-control. Considering that the improvement of the participants with low trait self-control in the disorderly condition was almost the same as that of people with high trait self-control in both the orderly and disorderly conditions, it was reasonable to infer that the orderly environment increased the persistence of the participants with low trait self-control, whereas the disorderly environment did not decrease the persistence. This was a new perspective for the environmental orderliness effect, as previous studies usually explained this effect on state self-control, assuming that a disorderly environment depleted self-control resources (Chae and Zhu, 2014). The current study suggested that an orderly environment could also increase the available self-control resources. However, for the participants with high trait self-control, the available self-control resources for the breath-holding task remained stable and were not affected by the environmental orderliness.

Another result worth discussing was that participants with low trait self-control showed more persistence than those with high trait self-control, especially in the orderly environment condition. This result seemed to contrast the expectation that participants with high trait self-control should have a better performance in the breath-holding task. To explain the results, first, we need to distinguish between the concept of “total self-control resources” and that of “available self-control resources.” The participants with high trait self-control had more total self-control resources; however, they could intentionally conserve the resources for the possible near future self-control task, consequently, providing only limited available resources for the breath-holding task (Muraven et al., 2006). Therefore, their persistence was reduced compared to the participants with low trait self-control in the orderly environment condition.

Previous research showed that when the participants regarded the target task as unworthy or they had near future self-control task, they could intentionally conserve the self-control resources after the depletion task, therefore performing poorly (Muraven et al., 2006; Dewall et al., 2011). However, deciding whether to exert self-control on the current task (e.g., breath-holding task), namely, managing the limited resources, itself is a form of self-control, which also deplete the self-control resources; as a result, given some circumstances, depleted individuals might perform better on certain tasks than non-depleted individuals (Dewall et al., 2011). Therefore, for the participants with low trait self-control, the improvement of performance on the breath-holding task does not necessarily suggest that the disorder did not deplete the self-control resources, as the depletion could be caused by the effort of managing the self-control resources instead of exerting self-control. Assuming this perspective, the participants with high trait self-control showed better self-control capacity than those with low trait self-control, as they could allocate the resources and keep their performance stable independently of the orderly or disorderly condition. In contrast, the participants with low trait self-control were vulnerable to the environmental orderliness.

In summary, Experiment 1 demonstrated that an orderly environment was more beneficial than a disorderly one in improving participants' persistence in the breath-holding task, which was in line with the environmental orderliness main effect hypothesis. More importantly, trait self-control could moderate the effect of environmental orderliness on state self-control, which supported the moderating hypothesis. Experiment 2 would explore whether the influence of environmental orderliness on creative thinking was also moderated by trait self-control.

## Experiment 2

Previous studies have shown that the manipulation of real environmental orderliness and conceptual picture priming had the same effect on state self-control and creative thinking, specifically, order induced better state self-control than disorder both in real environmental manipulations (Vohs et al., 2013) and picture-priming manipulation (Fan et al., 2012). Similarly, exposing the participants to an orderly or disorderly room, environmental disorder was more beneficial to creative thinking (Chen et al., 2013; Vohs et al., 2013), and environmental orderliness primed by pictures also had the same effect (Chen et al., 2013). Therefore, environmental orderliness has a stable effect on creative thinking, whether it is primed by pictures or manipulated by a real environment. To increase the diversity of the manipulation paradigm, Experiment 2 asked participants to observe and describe pictures to prime environmental orderliness. At the same time, the AUT was used to measure creative thinking and test our hypothesis. AUT, as a common divergent thinking task, requires individuals to list as many creative uses of a common object as possible. It requires individuals to think about and solve problems from multiple perspectives (Friedman and Förster, 2001; Vohs et al., 2013; Wang et al., 2018).

### Methods

#### Design and Participants

This experiment used a 2 (environmental orderliness: order vs. disorder) ×2 (trait self-control: high self-control vs. low self-control) between-subjects design. A total of 153 college students filled out the Chinese version of Brief SCS, and as in Experiment 1, a median split was used to sort participants into high vs. low self-control groups. A total of 124 participants (101 females; *M*_*age*_ = 20.38 years, *SD* = 1.28) volunteered to take part in the subsequent experiment, including 65 high trait self-control and 59 low trait self-control participants. There was a significant difference in Brief SCS scores between the high trait self-control group (*M*_high_ = 3.36, *SD* = 0.24) and the low trait self-control group (*M*_low_ = 2.55, *SD* = 0.25), *t*_(122)_ = −18.25, *p* < 0.001, Cohen's *d* = 3.28.

#### Procedure and Materials

**Figure 4:**
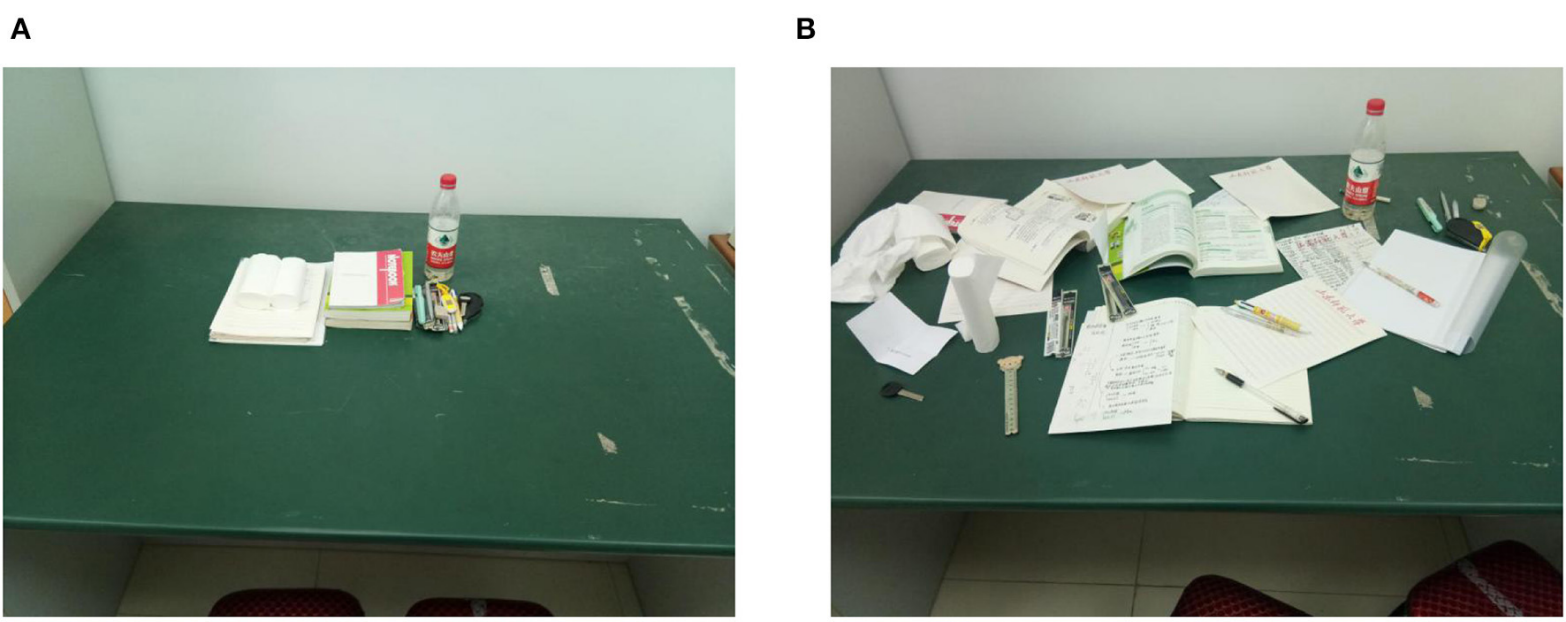
Illustration of the stimuli of Experiment 2: environmental order **(A)** and disorder **(B)** pictures.

Similar to Experiment 1, participants with high and low trait self-control were invited to participate in the follow-up experiment. Upon arrival, they were randomly assigned to the orderly or disorderly condition. In the orderly condition, a picture showed a desk with items placed in a well-organized manner. Instead, in the disorderly condition, the same items were scattered on the table in the picture (Figure 4). The participants were asked to look at the picture and imagine that they were in the corresponding scene (Fan et al., 2012; Chen et al., 2013). To increase their involvement, the participants were asked to describe the picture. After 3 min (Chen et al., 2013), they completed the AUT. The participants were asked to list as many “uncommon, creative, and unusual” uses of tires as possible within 3 min, and then circled two of the most creative uses (Silvia et al., 2008). Then, two questions were asked (i.e., “How messy is the scene in the picture? 1 = not at all, 7 = very messy” and “Do you think that the scene in the picture is orderly? 1 = not at all, 7 = very orderly”) to check whether environmental orderliness had been primed successfully (Chen et al., 2013). An index for perceived environmental orderliness was created by averaging the score of the two items (Cronbach's α = 0.91). Finally, participants were asked to guess the purpose of the experiment, fill in the demographic variables, and were debriefed and thanked.

#### Scoring Creativity

Participants' ideas were scored by the subjective scoring method (Silvia et al., 2008). Five raters, blind to condition, rated ideas separately from the other raters based on the scoring criteria (uncommon, remote, and clever) adopted by Wilson et al. (1953). Each response received a rating on a five-point scale (1 = low creativity, 3 = medium creativity, 5 = high creativity). The mean score of five raters (Cronbach's α = 0.85) was taken as the creative score for each response. Three creative indicators were generated: the average creativity score, the number of highly creative ideas (score more than 3 points), and Top 2 score (the mean score of the responses that participants themselves chose as the two best responses) (Silvia et al., 2008; Vohs et al., 2013).

### Results

The data were analyzed using SPSS 21.0. Two participants' data were excluded from the final analysis because the number of their highly creative ideas exceeded the mean of ±3 standard deviations. The final valid number was 122, including 35 high trait self-control participants and 29 low trait self-control participants in the orderly condition, and 29 high trait self-control participants and 29 low trait self-control participants in the disorderly condition. Three measurement indices of creative thinking under different conditions were analyzed.

#### Manipulation Check

The perception of environmental orderliness differed across conditions, *t*_(120)_ = −22.68, *p* < 0.001, Cohen's *d* = 4.11. The participants in the disorderly condition perceived the scene as more disorganized (*M*_disorder_ = 5.92, *SD* = 0.94) than those in the orderly condition (*M*_order_ = 2.07, *SD* = 0.94).

#### AUT: Moderation of Trait Self-Control

We hypothesized that trait self-control would moderate the influence of environmental orderliness on creative thinking. A two-factor MANOVA was performed on the average creativity score, the number of highly creative ideas, and Top 2 score.

##### The average creativity score

**Figure 5:**
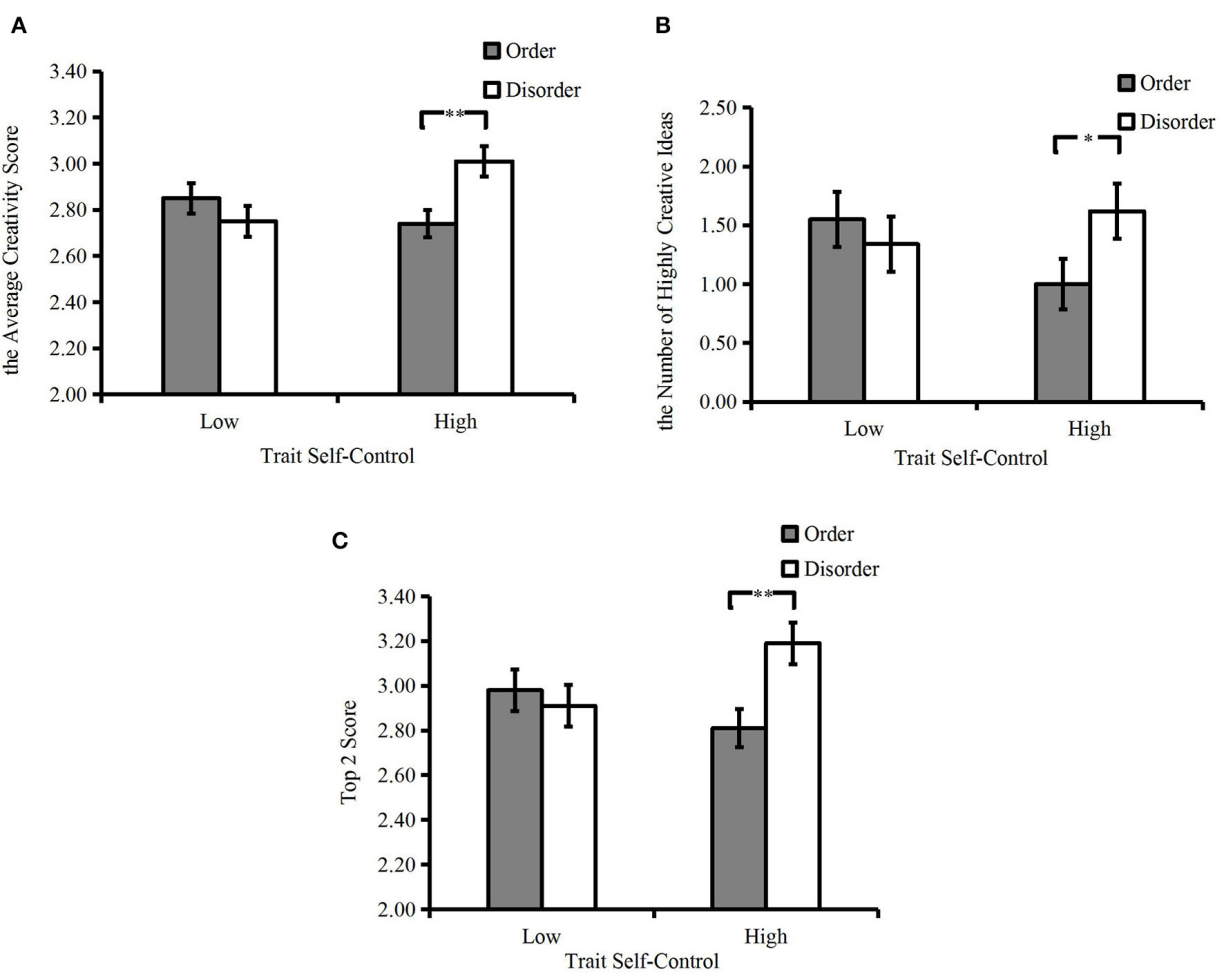
Experiment 2: participants' creative thinking performances (error bars represent standard errors; *p < 0.05; **p < 0.01).

Our moderating prediction was supported by the significant interaction between environmental orderliness and trait self-control, *F*_(1, 118)_ = 8.44, *p* = 0.004, $\eta_{p}^{2}$ = 0.07. Specifically, the average creativity scores of the participants with high trait self-control in the disorderly condition (*M*_disorder_ = 3.01, *SD* = 0.42) were significantly higher than that of those in the orderly condition (*M*_order_ = 2.74, *SD* = 0.33), *F*_(1, 118)_ = 9.32, *p* = 0.003, $\eta_{p}^{2}$ = 0.07; there was no difference in the average scores of low self-control participants between the two conditions (*M*_order_ = 2.85, *SD* = 0.35; *M*_disorder_ = 2.75, *SD* = 0.31), *F*_(1, 118)_ = 1.21, *p* > 0.05, $\eta_{p}^{2}$ = 0.01. In addition, there was no difference between the average creativity scores in two different environmental orderliness conditions, *F*_(1, 118)_ = 1.73, *p* > 0.05, $\eta_{p}^{2}$ = 0.01. There was also no difference in the scores between different trait self-control participants, *F*_(1, 118)_ = 1.23, *p* > 0.05, $\eta_{p}^{2}$ = 0.01 (see Figure 5).

##### The number of highly creative ideas

The pattern of interaction in the number of highly creative ideas was consistent with that of the previous indicator, *F*_(1, 118)_ = 3.28, *p* = 0.073, $\eta_{p}^{2}$= 0.03. Specifically, for the participants with high trait self-control, those in the disorderly condition generated more highly creative ideas (*M*_disorder_ = 1.62, *SD* = 1.32) than the participants in the orderly condition (*M*_order_ = 1.00, *SD* = 1.08), *F*_(1, 118)_ = 3.87, *p* = 0.052, $\eta_{p}^{2}$ = 0.03. However, there was no difference in the number of highly creative ideas of the participants with low trait self-control between the two conditions (*M*_order_ = 1.55, *SD* = 1.40, *M*_disorder_ = 1.34, *SD* = 1.23), *F*_(1, 118)_ <1, *p* > 0.05, $\eta_{p}^{2}$ = 0.003. There was also no difference between the number of highly creative ideas in the two different environmental orderliness conditions, *F*_(1, 118)_ <1, *p* > 0.05, $\eta_{p}^{2}$ = 0.01. Besides, there was also no difference in the number between different trait self-control participants, *F*_(1, 118)_ <1, *p* > 0.05, $\eta_{p}^{2}$ = 0.003 (see Figure 5).

##### Top 2 score

The significant interaction in Top 2 scores also supported our moderating prediction, *F*_(1, 118)_ = 6.03, *p* = 0.016, $\eta_{p}^{2}$ = 0.05. The results showed that the scores of the participants with high trait self-control in the disorderly condition (*M*_disorder_ = 3.19, *SD* = 0.62) were higher than in the orderly condition (*M*_order_ = 2.81, *SD* = 0.41), *F*_(1, 118)_ = 9.03, *p* = 0.003, $\eta_{p}^{2}$ = 0.07. In contrast, for the participants with low trait self-control, the Top 2 scores showed no difference between the two conditions (*M*_order_ = 2.98, *SD* = 0.53, *M*_disorder_ = 2.91, *SD* = 0.43), *F*_(1, 118)_ <1, *p* > 0.05, $\eta_{p}^{2}$= 0.002. In addition, the result also revealed that the participants in the disorderly environment (*M*_disorder_ = 3.05, *SD* = 0.55) marginally had higher Top 2 scores than those in the orderly one (*M*_order_ = 2.89, *SD* = 0.47), *F*_(1, 118)_ = 2.89, *p* = 0.092, $\eta_{p}^{2}$= 0.02. However, there was no difference in Top 2 scores between different trait self-control participants, *F*_(1, 118)_ <1, *p* > 0.05, $\eta_{p}^{2}$= 0.004 (see Figure 5).

#### AUT: Moderation of Environmental Orderliness

There was no main effect of trait self-control in these three indicators. In order to better understand the relationship between trait self-control and creative thinking, as kindly suggested by one of our reviewers, we further compared the creative thinking performance of high and low trait self-control participants, respectively, in the condition of order and disorder. The results showed that in the disorderly environment, the high trait self-control group had higher average creativity score [*M*_high_ = 3.01, *SD* = 0.42; *M*_low_ = 2.75, *SD* = 0.31, *F*_(1, 118)_ = 7.71, *p* = 0.006, $\eta_{p}^{2}$ = 0.06] and Top 2 score [*M*_high_ = 3.19, *SD* = 0.62; *M*_low_ = 2.91, *SD* = 0.43; *F*_(1, 118)_ = 4.62, *p* = 0.034, $\eta_{p}^{2}$ = 0.04] than the low trait self-control group, but not for the number of highly creative ideas [*M*_high_ = 1.62, *SD* = 1.32; *M*_low_ = 1.34, *SD* = 1.23, *F*_(1, 118)_ <1, *p* > 0.05]. In the orderly environment, low trait self-control participants produced more creative ideas than those with high trait self-control [*M*_low_ = 1.55, *SD* = 1.40; *M*_high_ = 1.00, *SD* = 1.08; *F*_(1, 118)_ = 3.05, *p* = 0.083, $\eta_{p}^{2}$ = 0.03]. However, there was no difference in the average score and Top 2 score between these two groups, *F*s <1, *p*s > 0.05.

The results showed an emerging pattern that under the condition of disorder environment, the creative thinking of high trait self-control participants was better; under the condition of order environment, the creative thinking of low trait self-control participants was better. However, the results of the statistical analysis showed that the pattern was not consistent for the three indices of creative thinking, especially for low trait self-control participants, all the three indices were not significant, which suggested that the moderating effect of environmental orderliness was not as reliable and stable as the moderating effect of trait self-control.

## Discussion

The results of Experiment 2 showed that three creative thinking indicators (the average creativity score, the number of highly creative ideas, and Top 2 score) consistently demonstrated that trait self-control could moderate the effect of environmental orderliness on creative thinking. This result supported our hypothesis and the compensation matching effect expectation. Specifically, for the participants with high trait self-control, compared with the orderly environment, the disorderly environment had a positive influence on creative thinking, whereas the creative thinking of those with low trait self-control was not affected by environmental orderliness. Therefore, we validated the moderating model (Figure 1). Three indicators revealed the same moderating direction, which showed that this moderation model was stable.

In addition, compared with previous findings showing that the participants in the disorderly environment performed better in creative thinking than those in the orderly environment (Chen et al., 2013; Vohs et al., 2013), among the three indices of creative thinking, only the Top 2 score results showed the trend according to which creative thinking improved in the disorderly condition compared to the orderly condition; however, this difference was not statistically significant. Therefore, the results did not support the main effect hypothesis of environmental orderliness, which rather suggested that the effect of environmental orderliness has the boundary condition of individual differences.

We tried to explain this moderating effect of trait self-control from the perspective of a resource model of self-control and the model of the dual pathway to creativity. First, the integrated model of the interaction between self-control resources and cognitive resources argues that ego depletion inhibits the exertion of executive control, making it difficult to allocate and transfer resources for a specific cognitive task. Therefore, ego depletion will have a negative impact on the cognitive processing (Li, 2013). Second, according to the model of the dual pathway to creativity, cognitive flexibility and cognitive persistence are the two main ways by which some situational or trait variables could affect individuals' creative thinking. Among them, cognitive persistence is needed to eliminate the interference of irrelevant information, allowing one to focus on the current task and conduct a systematic search, which depletes more resources than cognitive flexibility (De Dreu et al., 2008; Teng et al., 2018). Therefore, we thought that self-control resource depletion might have a negative impact on cognitive persistence; accordingly, compared with an orderly environment, a disorderly environment that could cause resource depletion was detrimental for cognitive persistence. Furthermore, we speculated that trait self-control could moderate the influence of environmental orderliness on creative thinking, which was mainly realized by influencing the available self-control resources, and further influencing the dual pathway of creative thinking (cognitive persistence and cognitive flexibility) asymmetrically. The participants with low trait self-control have fewer self-control resources (Baumeister, 2016). Compared with an orderly environment, a disorderly environment depletes part of their resources, which leads to their worse cognitive persistence. However, a disorderly environment can improve cognitive flexibility (Chen et al., 2013). On the whole, the effects of cognitive persistence and flexibility counteracted each other. Therefore, there was no difference in the creative thinking of the participants with low trait self-control in the conditions of order and disorder. On the one hand, although environmental disorder also depleted part of their resources, the cognitive persistence of the participants with high trait self-control was not significantly impaired by the condition of disorder, because they had more reserves of self-control resources (Baumeister, 2016), which were enough to compensate for the resource depletion caused by the disorderly environment. On the other hand, environmental disorder could improve cognitive flexibility (Chen et al., 2013); thus, it was more beneficial to the creative thinking of the participants with high trait self-control.

The results further illuminate the effect of environmental orderliness on creative thinking from the perspective of the dual pathway of creativity. Based on the results, we believe that environmental orderliness affected creative thinking by influencing the dual pathway of creative thinking. Environmental disorder did not only improve cognitive flexibility (Chen et al., 2013) but also depleted self-control resources and inhibited cognitive persistence; therefore, it was beneficial for the creative thinking of individuals with high trait self-control but not for those with low trait self-control. Previous studies had only examined the role of cognitive flexibility without considering the role of cognitive persistence (Chen et al., 2013). This experiment offers the new perspective of cognitive persistence to understand the influence of environmental orderliness on creative thinking.

## General Discussion

An orderly environment has a positive influence on individuals' state self-control behaviors, whereas a disorderly environment is beneficial to creative thinking. In two experiments, we investigated whether trait self-control could moderate this dual effect of environmental orderliness. Our moderating hypotheses were supported: the results revealed that an orderly environment was only beneficial to behavioral self-control for individuals with low trait self-control, whereas a disorderly environment only had a positive effect on the creative thinking of those with high trait self-control. The compensation matching effect expectation was supported; that is, individuals with high and low trait self-control need a matching level of environmental orderliness to achieve the best overall effect of orderliness on self-control behaviors and creative thinking. Specifically, matching individuals with low trait self-control with order can have a positive influence on their behavioral self-control without impairing creative thinking; on the other hand, matching those with high trait self-control with disorder can be beneficial to their creative thinking without impairing their self-control behaviors.

The current research makes several theoretical contributions. First, this study introduces trait self-control as a moderator of individual differences, expanding the boundaries of the environmental orderliness effect. Previous studies have shown that environmental orderliness affects both self-control (Fan et al., 2012; Vohs et al., 2013; Chae and Zhu, 2014) and creative thinking (Chen et al., 2013; Vohs et al., 2013; Zheng et al., 2016). Our study explored the moderating effects on both state self-control behaviors and creative thinking and identified a new boundary condition for the dual effect; that is, the impact of environmental orderliness on state self-control is only verified in individuals with low trait self-control, whereas its influence on creative thinking is only confirmed in those with high trait self-control.

Second, our findings are helpful to understand the common mechanism of the dual effect of environmental orderliness. Previous research discussed the mechanism by which environmental orderliness affects self-control and creative thinking separately, suggesting that the perception of a threat to personal control mediated the relationship between environmental orderliness and behavioral self-control (Chae and Zhu, 2014); cognitive flexibility and heuristic processing were assumed to be the mechanisms by which environmental orderliness influences creative thinking (Chen et al., 2013). These two types of mechanisms seem to be completely unrelated. However, the present study demonstrated that the available self-control resource is the common psychological mechanism of the dual effect by identifying the moderating role of trait self-control, which reflects the reserves of self-control resources. In fact, previous studies have found that environmental orderliness could affect self-control resources (Chae and Zhu, 2014), and resource depletion could affect state self-control (Baumeister et al., 1998; Muraven et al., 1998; Vohs and Heatherton, 2000) and creative thinking (Chiu, 2014), which also supports the idea that the available self-control resources are the common mechanism of the dual effect of environmental orderliness.

The new explanation of the available self-control resources as the psychological mechanism of the dual effect does not contradict the WIR model. On the contrary, it is a refinement and revision of this model. The reduction of the sense of personal control or the decrease of executive control proposed by the WIR model are the results of the reduction of available self-control resources. Furthermore, the explanation based on the available self-control resources could be used not only for the effect of environmental orderliness on self-control behaviors and creative thinking; it might also be used to explain other psychological and behavioral effects of environmental orderliness. For example, an orderly experience leads to global perceptual processing, whereas a disorderly experience leads to local perceptual processing (Li et al., 2019b), which may be because order increases the available self-control resources and disorder reduces the available self-control resources. A previous study has shown that the available self-control resources do play important roles in perceptual processing (Bruyneel and Dewitte, 2006).

In addition to its theoretical contributions, this research also has important practical implications concerning the shaping and cultivation of individuals' self-control and creative thinking. Previous research has demonstrated the benefits of order for self-control (Fan et al., 2012; Vohs et al., 2013; Chae and Zhu, 2014), and the benefits of disorder for creative thinking (Chen et al., 2013; Vohs et al., 2013). These results suggest that we could deliberately design the environment to improve self-control or creative thinking according to the needs of the specific task. For examples, when a task requires focus and perseverance, we could make the environment more orderly to improve personal state self-control. When a task requires creativity and flexibility, we could make the environment more disorderly to stimulate creative thinking. More importantly, the dual effect is moderated by trait self-control. There is a compensation matching effect, that is, individuals with high and low trait self-control need the matching level of environmental orderliness to achieve the best overall effect of orderliness on self-control behaviors and creative thinking. Specifically, matching low trait self-control individuals with order can have a positive influence on their behavioral self-control without impairing creative thinking; while, matching high trait self-control individuals with disorder can be beneficial to their creative thinking without impairing their self-control behaviors. The compensation matching effect could be applied in the fields of education and management to improve work efficiency. For example, for schoolchildren with low trait self-control, we could set a neat and orderly classroom to improve their self-control and support smooth teaching; on the other hand, for those who have high trait self-control and need creativity and flexibility (such as engineers of internet companies), we could stimulate creativity and flexibility by setting a moderately disorderly environment without compromising their focus and persistence.

This study focused on the moderating role of trait self-control on the dual effect of environmental orderliness and drew some meaningful conclusions. Nevertheless, there were some limitations. First, the common moderating effect of trait self-control provided evidence that the available self-control resources were the common psychological mechanism behind the dual effect, which could serve as the starting point for a deeper exploration of the specific psychological mechanism. However, how the available self-control resources influence creative thinking remains to be explored. Accordingly, future research could continue to explore in-depth how available self-control resources affect state self-control behaviors and creative thinking, as well as the specific mechanism of the moderating effect of trait self-control on this dual effect. Second, the limited sample size represents another limitation of this study. In future research, behavioral big data, such as those related to behavioral orderliness (e.g., Cao et al., 2018, 2019; Yao et al., 2019), could be used to classify trait self-control, this may increase the representativeness of the sample and add natural behavior observations to compensate for the weakness of the small sample size and social desirability bias of the measures based on self-report questionnaires. Third, we used real environment and conceptual priming to manipulate environmental orderliness in two experiments. Although previous studies showed that real environment or conceptual picture priming had the same effect on state self-control behaviors or creative thinking (Fan et al., 2012; Chen et al., 2013; Vohs et al., 2013) and the different manipulations increased the diversity of research paradigms, it also brought difficulties of comparing state self-control behavior and creative thinking under different paradigms. Future research should consider directly comparing the two dependent variables under the same paradigm. In addition, future research could explore whether other dependent variables, such as immoral and illegal behavior (Keizer et al., 2008) perceptual processing (Li et al., 2019b), are also moderated by trait self-control. If this hypothesis of a moderating role is still supported, it will provide more reliable evidence, which supports the suggestion that available self-control resources are the key mechanisms behind the psychological and behavioral effects of environmental orderliness.

## Conclusions

The present study showed that trait self-control moderates the effects of environmental orderliness on self-control behaviors and creative thinking. Order is beneficial to self-control, but it is effective only for individuals with low trait self-control; disorder is beneficial to creative thinking, but only for individuals with high trait self-control. These findings provide a new research perspective for the environmental orderliness literature, contribute to the understanding of the common mechanism of the dual effect, and have practical implications for the shaping and cultivation of individuals' self-control behaviors and creative thinking.

## Data Availability Statement

The datasets generated for this study are available on request to the corresponding author.

## Ethics Statement

The studies involving human participants were reviewed and approved by Institutional Review Board of the School of Psychology at the Shandong Normal University. The participants provided their written informed consent to participate in this study.

## Author Contributions

NL contributed to the conception and design of the research. ZL performed the experiments, analyzed the data, and wrote the first draft of the manuscript. NL, ZL, and SL revised the manuscript. All authors contributed to the article and approved the submitted version.

## Conflict of Interest

The authors declare that the research was conducted in the absence of any commercial or financial relationships that could be construed as a potential conflict of interest.
